# The Translational Coupling of Daidzein Reductase and Dihydrodaidzein Racemase Genes Improves the Production of Equol and Its Analogous Derivatives in Engineered Lactic Acid Bacteria

**DOI:** 10.1021/acssynbio.5c00532

**Published:** 2025-10-24

**Authors:** Susana Langa, José Antonio Curiel, Ángela Peirotén, José María Landete

**Affiliations:** Departamento de Tecnología de Alimentos, 54402 Instituto Nacional de Investigación y Tecnología Agraria y Alimentaria (INIA-CSIC), Crta de la Coruña Km7.5, 28040 Madrid, Spain

**Keywords:** 5-hydroxy-equol, 5-hydroxy-dehydroequol, translational coupling, dihydrodaidzein racemase, soy beverages, health

## Abstract

Equol (EQ) and its analogous derivatives 5-hydroxy-equol (5-OH-EQ) and 5-hydroxy-dehydroequol (5-OH-D-EQ) are isoflavones which benefit human health. They are produced from daidzein and genistein, respectively, in the gut by microorganisms harboring the genes daidzein reductase (*dzr*), dihydrodaidzein racemase (*ifc*A), dihydrodaidzein reductase (*ddr*) and tetrahydrodaidzein reductase (*tdr*). Since the production of these isoflavones is of interest due to their great-health benefits for humans, the heterologous expression of *dzr*, *ddr*, *tdr* and *ifc*A from *Slackia isoflavoniconvertenes* DSM 22006T in lactic acid bacteria (LAB) was used as a strategy to produce EQ, 5-OH-EQ and 5-OH-D-EQ in soy beverages. However, efficient production of these compounds was only demonstrated in two engineered *Limosilactobacillus fermentum* strains, and it is dependent on dihydrodaidzein racemase (DDRC). In order to increase the production of EQ and its analogous derivatives in different LAB species and genera, different strategies were performed with the *ifc*A gene. Translational coupling of *ifc*A and *dzr* genes (pNZ:TuR.dzr.ifcA) under the influence of a constitutive promoter improved the efficiency of production of EQ, 5-OH-EQ and 5-OH-D-EQ in the engineered LAB strains. The translational coupling of *ifc*A and *dzr* genes allowed the production of high concentrations of eq (111.15 ± 9.20–410.56 ± 24.15 μM), 5-OH-eq (71.00 ± 4.25 μM–148.22 ± 9.15 μM) and 5-OH-D-eq (111.15 ± 9.20–201.09 ± 7.65 μM) in soy beverages by different engineered LAB genera, such as *L. fermentum* INIA 584L, *Lactilactobacillus plantarum* WCFS1, and *Lactocaseibacillus paracasei* BL23. Translational coupling has allowed engineered Laboratories strains belonging to different genera, such as *L. fermentum*, *L. plantarum*, and *L. paracasei*, to produce high concentrations of EQ, 5-OH-EQ and 5-OH-D-EQ. Translational coupling could be exploited as a strategy for the efficient production of bioactive compounds.

## Graphical abstract

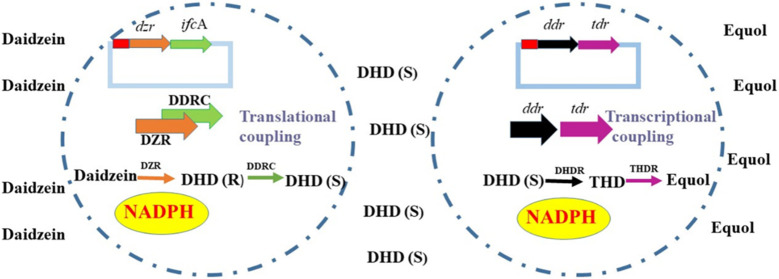

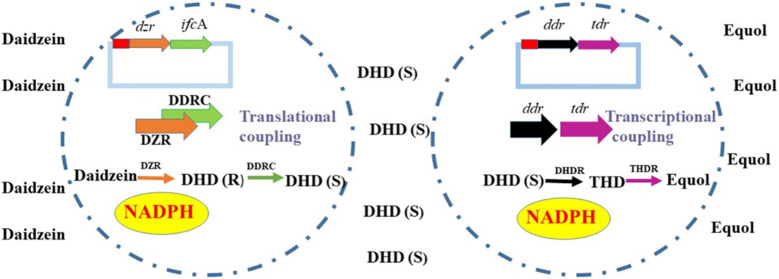

## Introduction

Soy intake is associated with beneficial effects on human health due to its content of isoflavones.^1–5^ However, there are many discrepancies between different studies concerning the beneficial effects of the isoflavones present in soy.^6,7^ For years, different research concerning the effect of isoflavones on health have associated these beneficial effects with the transformation of daidzein into equol (EQ).^8^ The population can be divided into nonequol-producing and equol-producing individuals, who are assumed to gain a beneficial effect from soy consumption as a consequence of the production of this bioactive isoflavone by their intestinal microbiota.^9^ More recently, the transformation of genistein into 5-hydroxy-equol (5-OH-EQ) has also been associated with beneficial effects.^10,11^

EQ is a phytoestrogen produced sequentially from daidzein via dihydrodaidzein and tetrahydrodaidzein, and 5-OH-EQ is produced from genistein via dihydrogenistein and tetrahydrogenistein. The production of these compounds is mainly limited to organisms which are difficult to work with such as *Slackia isoflavoniconvertenes* DSM 22006T, belonging to the Eggerthelaceae family.^7^ The genes involved in the production of EQ and 5-OH-EQ are the daidzein reductase gene (*dzr*), whose DZR protein is involved in the transformation of daidzein into dihydrodaidzein (DHD) and genistein into dihydrogenistein (DHG); the dihydrodaidzein reductase gene (*ddr*), whose DHDR enzyme is involved in the transformation of DHD and DHG into tetrahydrodaidzein (THD) and tetrahydrogenistein (THG), respectively; and finally the tetrahydrodaidzein reductase gene (*tdr*), whose THDR enzyme transforms THD into EQ and THG into 5-OH-EQ.^12,13^

In order to achieve the production of EQ and 5-OH-EQ in soy beverages, the *dzr*, *ddr* and *tdr* genes from *S. isoflavoniconvertenes* DSM 22006T were heterologously expressed in our laboratory in different LAB strains,^14^ with *Limosilactobacillus fermentum* INIA 584L and *L. fermentum* 832L being the only strains that showed an elevated production of EQ and 5-OH-EQ after incorporating the heterologous expression of dihydrodaidzein racemase (*ifc*A),^13,15^ whose DDRC enzyme transforms DHD (R) into DHD (S) and DHG (R) into DHG (S).^16^ Thus, the elevated production of EQ and 5-OH-EQ in engineered strains of *L. fermentum* INIA 584L and *L. fermentum* INIA 832L was due to the presence and activity of DDRC, which to date has only shown activity in these strains. However, the heterologous expression of *dzr*, *ifc*A, *ddr* and *tdr* in others engineered LAB strains showed low EQ production and did not show the production of 5-OH-EQ, moreover DDRC did not influence the production of EQ and 5-OH-EQ in these bacteria.^14^ On the other hand, the highest production of EQ and 5-OH-EQ by these *L. fermentum* strains has also been related to the elevated DHDR activity shown by the heterologous expression of *ddr*.^13^

More recently, the heterologous expression of the *dzr*, *ddr* and *ifc*A genes from *S. isoflavoniconvertenes* DSM 22006T in strains of *Escherichia coli* and LAB also demonstrated the transformation of genistein into a novel compound named 5-hydroxy-dehydroequol (5-OH-D-EQ) as a consequence of THG production from dihydrogenistein and subsequent hydration of the THG.^10,13^

As mentioned above, to date, only engineered *L. fermentum* 584L and *L. fermentum* INIA 832L have been able to produce high concentrations of EQ and analogous compounds. So, in this work, we propose the search for more engineered LAB strains for the production of high concentrations of EQ, 5-OH-EQ and 5-OH-D-EQ by engineered LAB strains using different strategies. Since DDRC seems to be responsible for the production of high concentrations of EQ, we propose different strategies with the *ifc*A gene such as the translational coupling of this gene. Translational coupling is the result of the stoichiometric synthesis of proteins by making the translation of a gene dependent on the gene immediately preceding it. Therefore, since translational coupling allows for faster and more efficient regulation of gene expression, we hypothesized that translational coupling of the *dzr* and *ifc*A genes would enhance the production of DHD (S) and DHG (S), and improve the production of equol and analogous compounds. For the assays of translational coupling of the *dzr* and *ifc*A genes, we first looked for a vector that could allow translational coupling. Several vectors from our collection were tested by our group. In this work, we will demonstrate the translational coupling of *mrfp* and *evoglow*.Pp1 genes in the vector pNZ:TuR.aFP.STOP.mCherry,^17^ previously built by our group. Subsequently, we will study the effect of translational coupling of the *dzr* and *ifc*A genes in the production of EQ and analogous compounds.

## Materials and Methods

### Bacterial Strains, Growth Conditions and Plasmids Used in This Study

The bacterial strains used in this study are *Lactococcus cremoris* MG1363,^18^ *Streptococcus thermophilus* INIA 468,^19^ *Lacticaseibacillus paracasei* BL23,^20^ *L. paracasei* INIA P272,^21^ *Lactiplantibacillus plantarum* WCFS1,^22^ *Lacticaseibacillus rhamnosus* INIA P540,^21^ *Limosilactobacillus reuteri* INIA P572,^23^ *L. fermentum* INIA 225L (this work), *L. fermentum* INIA 143L (this work), *L. fermentum* INIA 584L and *L. fermentum* INIA 832L.^24^ *L. cremoris* MG1363, *S. thermophilus* INIA 468 and their transformants were grown at 30 °C in M17 broth (Scharlab, Senmanat, Spain) supplemented with 0.5% glucose (Merck KGaA, Darmstadt, Germany) (GM17) under aerobic conditions. Lactobacilli strains and their transformants were routinely cultivated at 37 °C in MRS broth (BD Biosciences, Le Pont de Claix, France) under anaerobic conditions (10% H_2_, 10% CO_2_ and 80% N_2_. Whitley DG250 Anaerobic workstation, Don Whitley Scientific Ltd., Shipley, UK). Anaerobic conditions were checked periodically using a Resazurin Anaerobic Indicator (cat. no. BR0055B; Thermo Fisher Scientific, Waltham, MA, USA). For the growth of transformed strains, a final concentration of 5 μg/mL of chloramphenicol (Merck) was used.

*Bifidobacterium pseudocatenulatum* INIA P815 was grown in MRS broth (BD Biosciences) supplemented with 0.5 g/L l-cysteine (Merck) at 37 °C for 48 h under anaerobic conditions.

The plasmids used in this study are listed in Table 1, and chloramphenicol (5 μg/mL, Merck KGaA, Darmstadt, Germany) was used for the growth and selection of transformant strains with this plasmid.

**Table 1:** Plasmids Used in This Work

plasmids	characteristic relevant	refs
pNZ:TuR	pNZ8048 in which Pnis was replaced with the promoter of elongation factor Tu from *L. reuteri* CECT925	^26^
pNZ:TuR.aFP	pNZ:TuR harboring *ewoglow*Pp1 gene	^26^
pNZ:TuR.mCherry	pNZ:TuR harboring *mrfp* gene	^17^
pNZ:TuR.aFP.STOP.mCherry	pNZ:TuR harboring *evoglowPp*1 and *mrfp* genes cloned together with stop condon between both genes	^17^
pNZ:TuR.ΔATG.aFP.STOP.mCherry	pNZ:TuR.aFP.STOP.mCherry without the ATG from evoglowPp1	this work
pNZ:TuR.dzr	pNZ:TuR harboring *dzr* gene	^24^
pNZ:TuR.tdr.ddr	pNZ:TuR harboring *ddr* and *tdr* *S. isoflavoniconvertens* DSM 22006T	^14^
pNZ:TuR.ifcA	pNZ:TuR harboring *ifc*A from *S. isoflavoniconvertens* DSM 22006T	^14^
pNZE:TuR.dzr	pNZE:TuR harboring *dzr* from *S. isoflavoniconvertens* DSM 22006T	^15^
pNZ:TuR.dzr.ifcA	pNZ:TuR harboring *dzr* and *ifc*A gene	This work

### Effect of the Heterologous Expression of *ifc*A on the Production of EQ and Analogous Compounds by Engineered LAB Strains

The 11 LAB strains harboring pNZ:TuR.dzr and pNZ:TuR.tdr.ddr were inoculated at 1% (v/v) in BHI medium supplemented with daidzein (50 mg/L; 196,77 μM) and in BHI medium supplemented with genistein (50 mg/L; 185.02 μM) and incubated for 72 h at 30 or 37 °C in anaerobic conditions. Different LAB strains harboring pNZ:TuR, which did not show the production of EQ or analogous compounds, were used as the negative control. Subsequently, the samples were frozen at −30 °C until their extraction and subsequent analysis. Three independent experiments (biological replicas) were performed.

Later, with the aim of studying the effect of the heterologous expression of *ifc*A in the production of EQ, 5-OH-EQ and 5-OH-D-EQ, the tests were repeated with the incorporation of the same strains harboring pNZ:TuR.ifcA. The 11 LAB strains harboring pNZ:TuR.dzr, pNZ:TuR.tdr.ddr and pNZ:TuR.ifcA were inoculated in BHI medium supplemented with daidzein under the same conditions mentioned above. After the incubation times, the samples were frozen at −30 °C until their extraction and subsequent analysis.

### Effect of Heterologous Expression of *ifc*A and *dzr* in Different Vectors and in the Same Cell in EQ Production by Engineered LAB Strains

#### Construction of LAB Strains Harboring *dzr* and *ifc*A in Different Vectors

The objective was to develop a strain of LAB containing the two genes *dzr* and *ifc*A. Thus, the 11 LAB strains harboring pNZ:TuR.ifcA were transformed with pNZE:TuR.dzr (Langa et al.) as described by Landete et al. Transformants harboring both plasmids were selected with erythromycin and chloramphenicol (5 μg/mL, Merck). The presence of pNZE.TuR.dzr was checked by means of PCR.

The stability of vectors in the different Laboratories was assayed by growing the cells in nonselective media for approximately 100 generations and plating daily onto nonselective GM17 or MRS agar plates. Loss of the integrated plasmid was determined by replica-plating colonies on GM17 or MRS agar plates with or without an antibiotic (erythromycin or chloramphenicol). Moreover, the presence of plasmids was monitored by PCR.

#### Effect of Heterologous Expression of *ifc*A and *dzr* in the Same Cell in EQ Production

*L. cremoris* MG1363, *L. paracasei* BL23, *L. plantarum* WCFS1, *L. fermentum* INIA 143L, *L. fermentum* INIA 584L and *L. fermentum* INIA 849L strains harboring both plasmids (pNZE.TuR.dzr and pNZ:TuR.ifcA) were incubated with these LAB strains harboring pNZ:TuR.tdr.ddr in BHI medium (BD; Becton, Dickinson & Co., Le Pont de Claix, France) supplemented with daidzein (50 mg/L; 196,77 μM). The strains were added at 1% (v/v) and they were incubated for 72 h at 30 or 37 °C in anaerobic conditions. After the incubation time, the samples were frozen at −30 °C until their extraction and subsequent analysis.

### Translational Coupling of *dzr* and *ifc*A to Improve the Production of EQ and Analogous Compounds by Engineered Lactic Acid Bacteria

#### Demonstration of Translational Coupling in pNZ:TuR.aFP.STOP.mCherry

To confirm the existence of the translational coupling of ORF2 (*mrfp*) in pNZ:TuR.aFP.STOP.mCherry, the ATG of ORF1 (*evoglow*-Pp1) was eliminated. In order to do this, the *evoglow-*Pp1 gene was amplified with the forward primers F.Δ.ATG.aFP, where the ATG initiation of evoglow.Pp1 translation was change by ATA, and the reverse primer R-aFP, using the plasmid pNZ:TuR.aFP as the template. Later, the PCR products and pNZ:TuR.aFP.STOP.mCherry were digested with *BsrG1*-HF and *Xba*I and ligated. Later, the ligation mixtures harboring pNZ:TuR.ΔATG.aFP.STOP.mCherry were used to transform *L. cremoris* MG1363 by electroporation,^25^ and the transformants were selected with chloramphenicol (5 μg/mL, Sigma-Aldrich) and checked by restriction mapping and sequencing of the inserted fragment. Subsequently, a proteomic analysis was performed to study the production of EvoglowPp1 and mCherry. *L. cremoris* MG1363 harboring pNZ:TuR.ΔATG.aFP.STOP.mCherry, pNZ:TuR.aFP, pNZ:TuR.mCherry and pNZ:TuR.aFP.STOP.mCherry (Table 1 and Figure 1) were grown on GM17 agar plates supplemented with chloramphenicol to produce a bacterial lawn. *L. cremoris* MG1363 harboring these vectors were recovered from grown bacterial lawns in solid media in 500 μL of PBS 1× using an inoculation loop handle. Then, the resuspended cultures were centrifuged at 6000*g* for 15 min, discarding the supernatants. Bacterial pellets (0.01 g per sample) were kept at −20 °C until use. Protein extraction from the pellets was performed using the Cellytic B Plus Kit (Merck KGaA, Darmstadt, Germany) which includes the CelLytic B Bacterial Lysis Reagent, lysozyme, benzoase and protein inhibitors.

**Figure 1:**
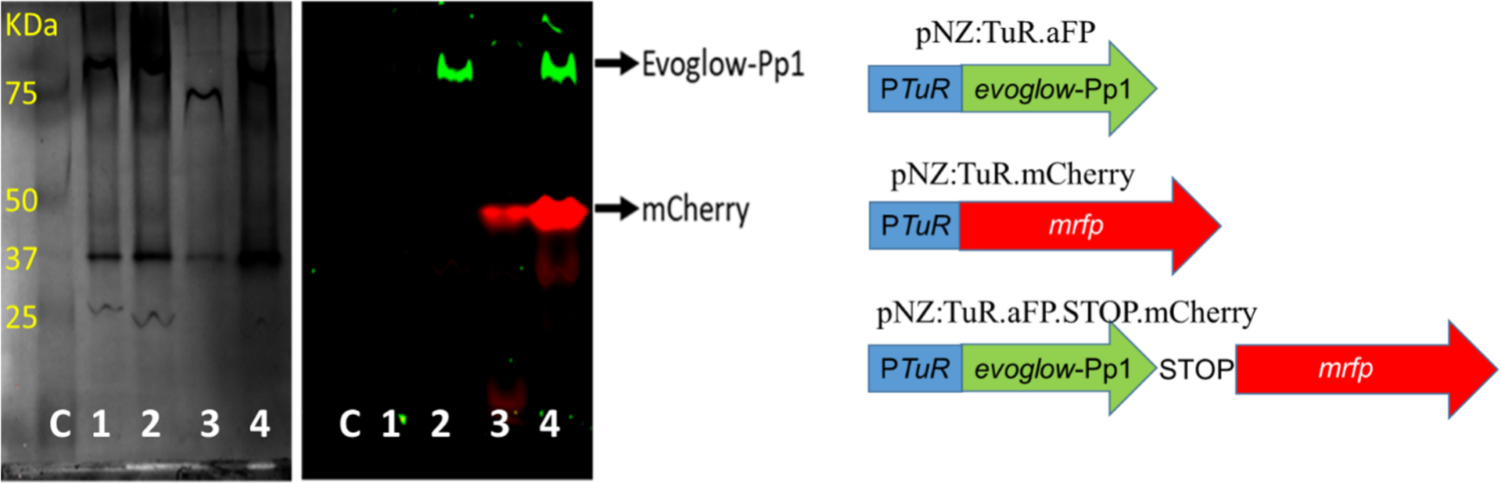
PAGE analysis under native conditions of protein extracts from parental and transformed strains of *L. cremoris* MG1363. Control parental strain (C) and transformed strains with pNZ:TuR.ΔATG.aFP.STOP.mCherry (1), pNZ:TuR.aFP expressing Evoglow-Pp1, pNZ:TuR.mCherry expressing mCherry (3) and pNZ:TuR.aFP.STOP.mCherry expressing Evoglow-Pp1 and mCherry separately (4). Fluorescence was checked for green (blue epi illumination and emission filter of 530/28 nm) and red (green epi illumination and emission filter of 605/50 nm) fluorescence using a ChemiDoc MP system.

After preparation of protein extracts, we carried out the PAGE protein analysis. For PAGE under native conditions, Mini-PROTEAN TGX Precast 8–16% polyacrylamide gradient gels were used. Protein samples (25 μL) were mixed with 25 μL native sample buffer 2×. Precision Plus Protein All Blue Pre-Stained Protein Standard (10–250 kDa) was loaded in every experiment. Electrophoresis was run initially at 60 V for 30–45 min and then at 90–120 V for 3 h using a native running buffer. Gel electrophoresis equipment (Mini-PROTEAN Tetra Cell) and reagents were purchased from Bio-Rad Laboratories (Madrid, Spain). After electrophoresis, the gels were placed in a ChemiDoc MP imaging system Bio-Rad Laboratories (Madrid, Spain) and the fluorescence was detected. Green fluorescent protein bands were detected with blue epi illumination and a 530/28 nm emission filter, whereas red fluorescent protein bands were checked using green epi illumination and a 605/50 nm emission filter. The Multichannel tool (Image Lab software) was used to overlay both green and red fluorescence images within the same gel.^17^

#### Identification of the Putative Translation Start Codons of the *mrfp* Gene in pNZ:TuR.aFP.STOP.mCherry

There are three ATGs in the reading phase and therefore three possible *mrfp* translation start codons (Figure 2). To identify which one(s) functioned as *mrfp* translation start codons in pNZ:TuR.aFP.STOP.mCherry, *mrfp* was amplified with the primers carrying a single ATG, and the other two “ATGs” were changed to “ATAs”. Three amplifications were performed, one for each ATG, using the primers F-CherryATG1, F-CherryATG2 and F-CherryATG3, and the reverse R-mCherry (Table 2), using the plasmid pNZ:TuR. mCherry as the template. The PCR products were digested with *Xba*I and *Sac*I and ligated into the corresponding restriction sites of pNZ:TuR.aFP. The ligation mixtures harboring pNZ:TuR.aFP.STOP.mfp1 (pATG1), pNZ:TuR.aFP.STOP.mfrp2 (pATG2) and pNZ:TuR.aFP.STOP.mrfp3 (pATG3) were used to transform *L. cremoris* MG1363 by electroporation,^25^ and the transformants were selected with chloramphenicol (5 μg/mL, Sigma-Aldrich) and checked by restriction mapping and sequencing of the inserted fragment. Later, the green and red fluorescence were analyzed by ChemiDoc according to Langa et al.^17^

**Figure 2:**
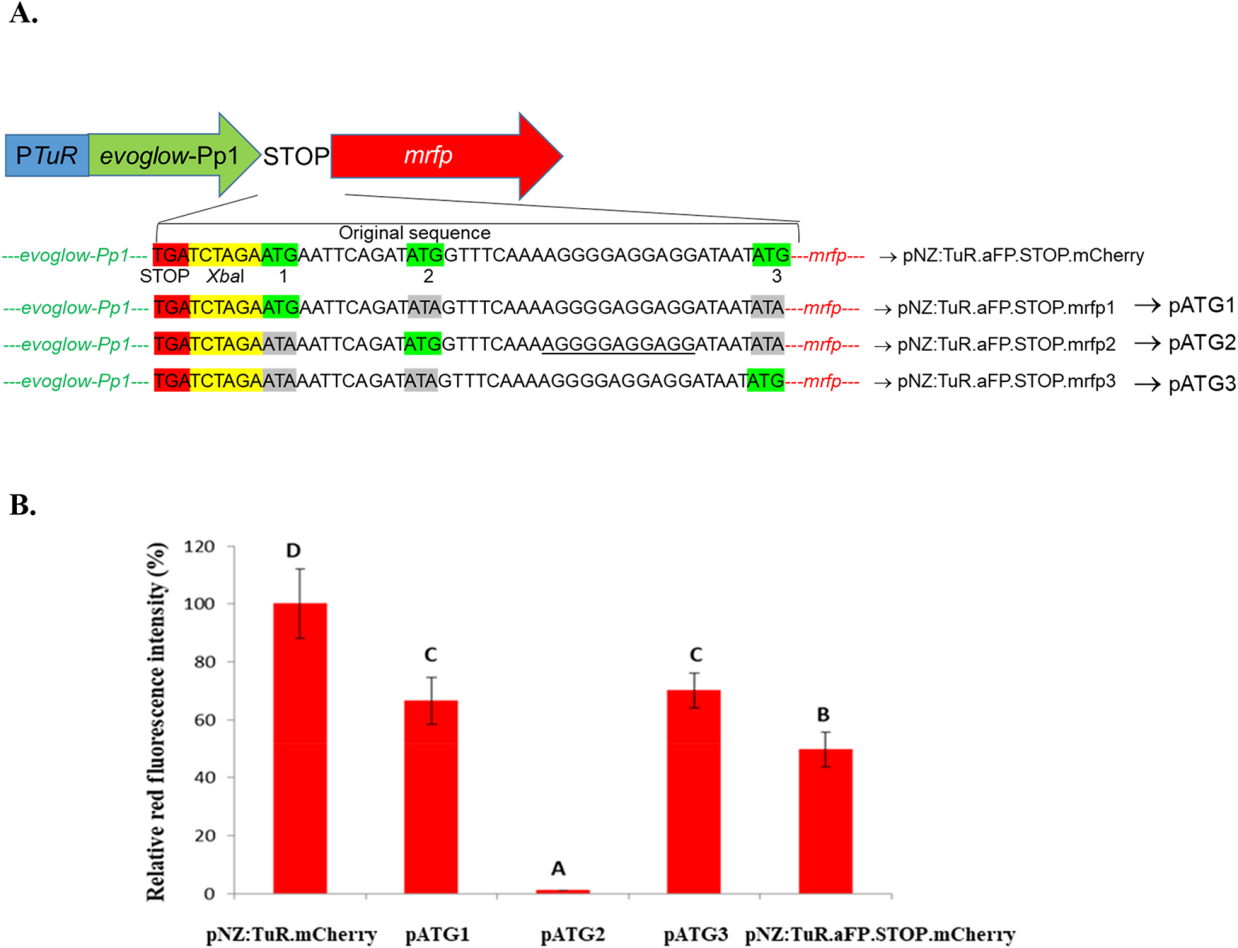
Plasmid with fluorescence protein genes used in this work with the original sequence of the initial *mrfp* fragment after the stop codon and with modifications of original sequence for determining the participation of ATGs in the start of mrfp translation (A). Relative red fluorescence intensity of pATG1 (pNZ:TuR.aFP.STOP.mrfp1), pATG2 (pNZ:TuR.aFP.STOP. mrfp2) and pATG3 (pNZ:TuR.aFP.STOP.mrfp3) was measured in relation to the original sequence pNZ:TuR.aFP.STOP.mCherry and pNZ:TuR.mCherry (B). Fluorescence was checked for red (green epi illumination and emission filter of 605/50 nm) fluorescence using a ChemiDoc MP system. The data presented are the means of replicates (*n* = 3) and error bars represent the standard deviation. Different superscripts indicate statistically significant differences (*P* < 0.01) in red fluorescence emission.

**Table 2:** List of Primers Used in This Work

primer	sequence (5′→ 3′)
F-CherryATG-3	TTTTCTAGAATAAATTCAGATATAGTTTCAAAAGGGGAGGAGGATAATATGGCGATTATC
F-CherryATG-2	TTTTCTAGAATAAATTCAGATATGGTTTCAAAAGGGGAGGAGGATAATATAGCGATTATC
F-CherryATG-1	TTTTCTAGAATGAATTCAGATATAGTTTCAAAAGGGGAGGAGGATAATATAGCGATTATC
R-mCherry	TTTGAGCTCTCATTTATATAATTCGTCCATGCCAC
F-ΔATG.aFP	GAAGTTGTACAATATGTATAAGGGTATGTCAGTCACCGAATCAGATGATCTGGCATTATACTTGTAAATTATCAGGAGGTTTTCATCCATAGTCAACGCAAAACTCCTGCAACTGATGG
R-aFP	TTTTTCTAGATCAGTGCTTGGCCTGGCCCTGCTG
F-ifcA	TTTTCTAGAATAAATTCAGATATAGTTTCAAAAGGGGAGGAGGATAATATGCTGCTCAAGGGCGAGTTTGCAGC
R-ifcA	TTTTCTAGACTACTCAGCGTCCACGTCGCAAACG

#### Construction of Plasmid pNZ:TuR.dzr.ifcA

The gene encoding dihydrodaidzein racemase (*ifc*A) from *S. isoflavoniconvertens* DSM 22006T was amplified by PCR using the primers F-ifcA and R-ifcA (Table 2). The forward primer introduced a sequence that allowed the translational coupling of the gene located upstream (*dzr*) with the gene located downstream (*ifc*A) in accordance with previous work by our group with pNZ:TuR.aFP.STOP.mCherry^17^ for the translational coupling shown between *evoglow*-Pp1 and *mrfp* in the present work (Figure 3). The PCR products were digested with *Xba*I and ligated into vector pNZ:TuR.dzr (Table 1) digested with *Xba*I. The ligation mixtures harboring pNZ:TuR.dzr.ifcA were transformed into *L. cremoris* MG1363 by electroporation^25^ and the transformants were selected with chloramphenicol (5 μg/mL, Merck), and the PCR with the primer F-pNZ8048 ^26^ and R-rac (Table 2). The plasmid was sequenced to verify the correct sequence of the *dzr* and *ifc*A genes. Later, *S. thermophilus* INA 468, *L. paracasei* BL23, *L. paracasei* INIA P272, *L. plantarum* WCFS1, *L. rhamnosus* INIA P540, *L. reuteri* INIA P572, *L. fermentum* INIA 225L, *L. fermentum* INIA 143L, *L. fermentum* INIA 832L and *L. fermentum* INIA 584L were transformed with pNZ:TuR.dzr.ifcA as described by Landete et al.^25^ and selected on GM17 or MRS plates with chloramphenicol. The stability of vectors in the different LAB strains was analyzed as mentioned above.

**Figure 3:**
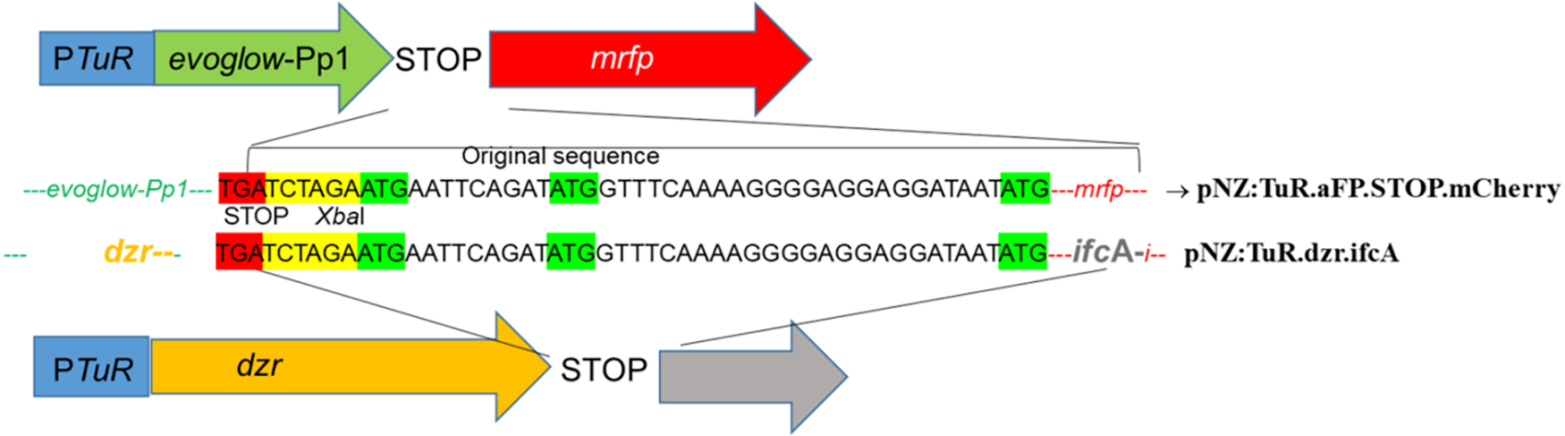
Construction of the vector pNZ:TuR.dzr.ifcA from pNZ:TuR.aFP.mCherry producing translational coupling between *dzr* and *ifc*A.

#### Effect of pNZ:TuR.dzr.ifcA on the Production of EQ and Analogous Compounds

To study the effect of the translational coupling of *dzr* and *ifc*A, LAB harboring pNZ:TuR.dzr.ifcA and pNZ:TuR.tdr.ddr were inoculated separately at 1% (v/v) in BHI medium supplemented with daidzein (50 mg/L; 196.77 μM) and in BHI medium supplemented with genistein (50 mg/L; 185.02 μM), then they were incubated for 72 h at 30 or 37 °C in anaerobic conditions. Subsequently, the samples were frozen at −30 °C until their extraction and subsequent analysis. We then compared the production of EQ and analogous compounds based on the different strategies used.

#### Production of EQ and Analogous Compounds in Soy Beverages with the Different Strategies Used with *ifc*A

*B. pseudocatenulatum* INIA P815, due to its ability to deglycosylate daidzin and genistein into daidzein and genistein, was coincubated separately with three combinations of *L. fermentum* INIA 584L strains: (1) *L. fermentum* INIA 584L pNZ:TuR.dzr, *L. fermentum* INIA 584L pNZ:TuR.ifcA and *L. fermentum* INIA 584L pNZ:TuR.tdr.ddr; (2) *L. fermentum* INIA 584L pNZE.TuR.dzr + pNZ:TuR.ifcA and *L. fermentum* INIA 584L pNZ:TuR.tdr.ddr; and (3) *L. fermentum* INIA 584L pNZ:TuR.dzr.ifcA and *L. fermentum* INIA 584L pNZ:TuR.tdr.ddr. *L. fermentum* INIA 584L strains harboring the different plasmids and *B. pseudocatenulatum* INIA P815 were grown in their respective media for 24 and 48 h, respectively, under anaerobic conditions. After, 5 mL of each culture was centrifuged and resuspended in 0.5 mL of the soy beverages (VegeDia, DIA, Spain). These suspensions were used to inoculate 10 mL of the same soy beverage with concentrations between 1 × 10^7^ and 1 × 10^8^ cfu/mL. The different inoculated soy beverages were incubated for 48 h at 37 °C under anaerobic conditions and samples of the fermented soy beverages were collected every 6 h. Three biological replicas of all fermentations were carried out. Noninoculated soy beverages were included as the control. These same tests were repeated with the same combination of vectors: (1) pNZ:TuR.dzr, pNZ:TuR.ifcA and pNZ:TuR.tdr.ddr; (2) pNZE.TuR.dzr + pNZ:TuR.ifcA and pNZ:TuR.tdr.ddr, and (3) pNZ:TuR.dzr.ifcA and pNZ:TuR.tdr.ddr with *L. plantarum* WCFS1 and *L. paracasei* BL23, which were coincubated with *B. pseudocatenulatum* INIA P815. In addition, the evolution of pH in the fermented soy beverage and the microbial behavior of different strains was performed as described by Langa et al.^15^

#### Extraction and Quantification of Isoflavones

For the quantification of isoflavones in culture media, bacterial suspensions were removed by centrifugation and isoflavones were extracted with diethyl ether and ethyl acetate^27^ and then analyzed by HPLC-ESI/MS.^28^

EQ, genistein, and daidzein were bought from LC Laboratories (Woburn, MA), and DHD and DHG from Toronto Research Chemicals (Toronto, Canada). There are no standard compounds of 5-OH-EQ and 5-OH-D-EQ. Therefore, 5-OH-EQ and 5-OH-D-EQ were quantified with the calibration curves of equol.

#### Statistical Analysis

At least three independent experiments (biological replicas) were performed in all experiments. The data was statistically analyzed using IBM Corp.’s (Armonk, NY, USA) SPSS Statistics 22.0 program. An ANOVA was used to analyze the data using a general lineal model (GLM), and Tukey’s test was used to compare the means with a 99% confidence interval.

## Results

### Effect of the Heterologous Expression of *ifc*A on EQ Production from Daidzein by Engineered LAB

Figure 4 (blue bars) and Table 1S show the production of equol from daidzein by 11 LAB strains which had been transformed with the plasmids pNZ:Tu.dzr and pNZ:TuR.tdr.ddr. These plasmids harbor the genes for *dzr* that encode the enzyme DZR, and the genes for *ddr* and *tdr* that encode the enzymes DHDR and THDR. Of the 11 LAB strains harboring pNZ:TuR.dzr and pNZ:TuR.tdr.ddr, we observed a group of LAB strains made up of *L. paracasei* INIA P272, *L. rhamnosus* INIA P540, *L. reuteri* INIA P572 and *S. thermophilus* INIA 461, which produced very low concentrations of equol (lower than 10 μM of equol). A second group of microorganisms, made up of *L. cremoris* MG1363, *L. paracasei* BL23, *L. plantarum* WCFS1, *L. fermentum* INIA P143, *L. fermentum* INIA 225L, which produced concentrations close to 20 μM of equol, and finally *L. fermentum* INIA 584L and *L. fermentum* INIA 832L which produced concentrations of nearly 40 μM of equol.

**Figure 4:**
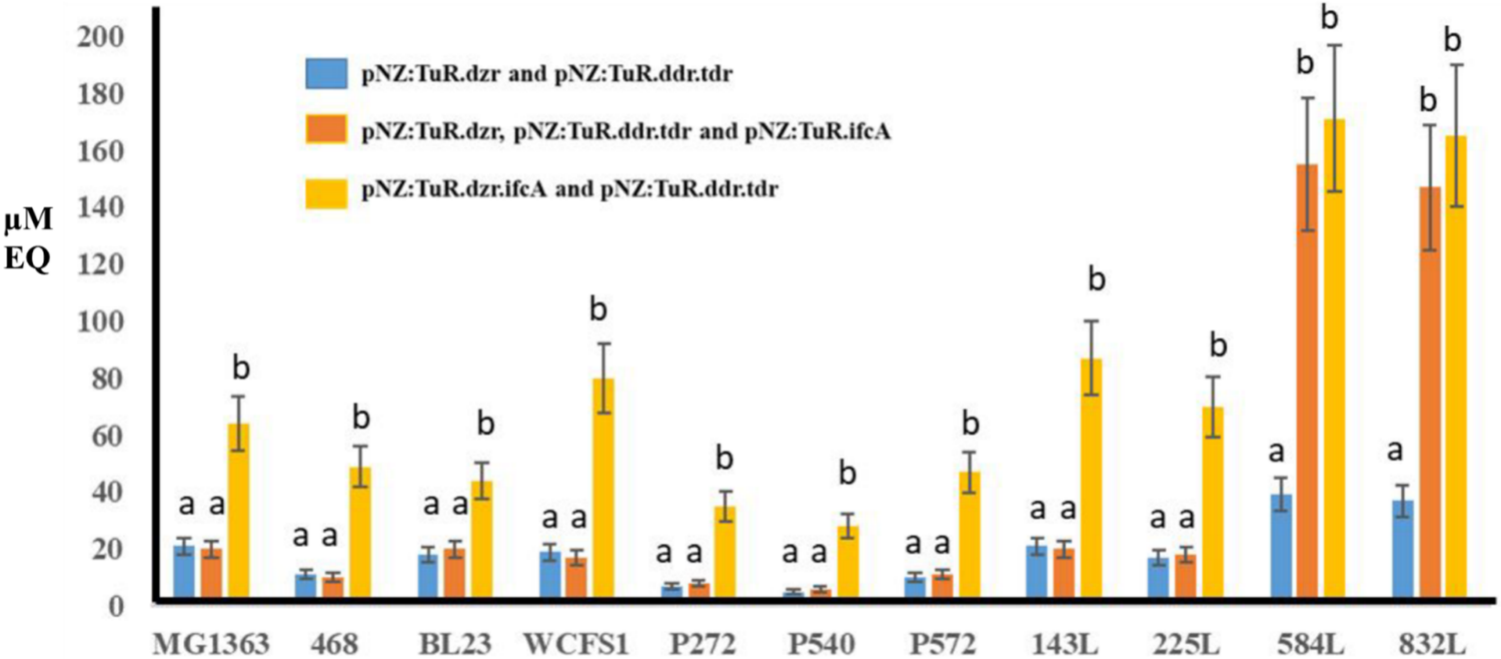
Equol production from daidzein after 72 h by LAB strains harboring the following combinations of plasmids: (i) pNZ:TuR.dzr and pNZ:TuR.tdr.ddr; (ii) pNZ:TuR.dzr, pNZ:TuR.tdr.ddr and pNZ:TuR.ifcA; (iii) pNZ:TuR.dzr.ifcA and pNZ:TuR.tdr.ddr. Different letters indicate significant difference at the level of *p* < 0.01.

Later, the 11 LAB strains were transformed with pNZ:TuR.ifcA, which contains the *ifc*A gene encoding DDRC. Subsequently, Laboratories strains harboring pNZ:TuR.ifcA were coincubated with the above-mentioned Laboratories strains harboring pNZ:TuR.dzr and pNZ:TuR.tdr.ddr in the presence of daidzein, and the equol production was analyzed (Figure 4, dark orange bars). The heterologous expression of *ifc*A from pNZ:TuR.ifcA only showed a significant effect (*p* < 0.01) on EQ production from daidzein in *L. fermentum* INIA 584L (155.20 ± 12.08 μM) and *L. fermentum* INIA 832L (147.45 ± 9.21 μM). Therefore, DDRC increased equol production 4 times (Figure 4 and Table 1S). On the other hand, the different LAB strains harboring pNZ:TuR did not show the production of EQ or any analogous compounds.

### Effect of the Heterologous Expression of *ifc*A on 5-OH-EQ Production from Genistein by Engineered LAB Strains

Figure 5 (blue bars) shows the production of 5-OH-EQ from genistein by strains harboring pNZ:TuR.dzr and pNZ:TuR.tdr.ddr was clearly lower than that of equol, and some strains, such as *L. paracasei* BL23, *L. paracasei* INIA P272, *L. rhamnosus* INIA P540 and *L. reuteri* INIA P572, were unable to produce 5-OH-EQ. The incorporation of the heterologous expression of *ifc*A (pNZ:TuR.ifcA) also failed to produce 5-OH-EQ by these strains (Figure 5, dark orange bars).

**Figure 5:**
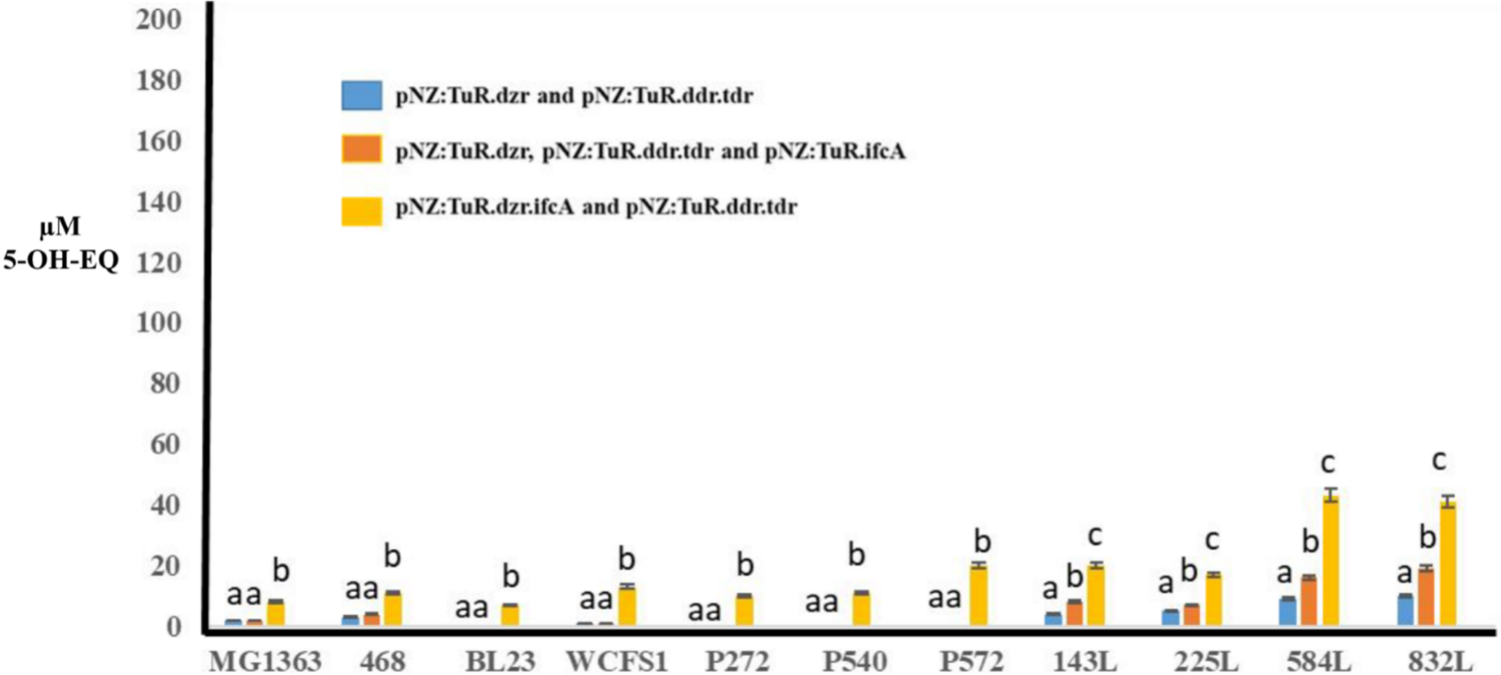
5-hydroxy-equol (5-OH-EQ) production from genistein after 72 h by LAB strains harboring the following combinations of plasmids: (i) pNZ:TuR.dzr and pNZ:TuR.tdr.ddr; (ii) pNZ:TuR.dzr, pNZ:TuR.tdr.ddr and pNZ:TuR.ifcA; (iii) pNZ:TuR.dzr.ifcA and pNZ:TuR.tdr.ddr. Different letters indicate significant difference at the level of *p* < 0.01.

On the other hand, *L. cremoris* MG1363, *S. thermophilus* INIA 461 and *L. plantarum* WCFS1 showed the production of very low concentrations of 5-hydroxy-equol, between 1 and 4 μM of this compound, and the incorporation of the heterologous expression of DDRC (pNZ:TuR.ifcA) did not show an increase in 5-OH-EQ production (Figure 4, dark orange bars) and Table 1S. However, the four *L. fermentum* strains, mainly *L. fermentum* INIA 584L and *L. fermentum* INIA 832L, showed a production of 5-OH-EQ between 4 and 10 μM, and the incorporation of the heterologous expression of DDRC (pNZ:TuR.ifcA) showed a significant (*p* < 0.01) increase in 5-OH-EQ production in the four engineered *L. fermentum* strains, with *L. fermentum* INIA 584L and *L. fermentum* INIA 832L reaching concentrations close to 20 μM with the presence of DDRC (Figure 5 and Table 2S).

### Effect of the Heterologous Expression of *ifc*A on 5-OH-D-EQ Production from Genistein by Engineered LAB Strains

Figure 6 (blue bars) and Table 3S show 5-OH-D-EQ production from genistein by Laboratories strains harboring pNZ:TuR.dzr and pNZ:TuR.tdr.ddr. The production of this compound varied from concentrations close to 5 μM, produced by engineered *L. paracasei* BL23, to concentrations close to 30 μM, produced by engineered *L. fermentum* INIA 584L. The incorporation of DDRC (pNZ:TuR.ifcA) showed a significant (*p* < 0.01) effect on the production of this compound in the 11 engineered LAB strains (Figure 6, dark orange bars). Engineered LAB strains increased 5-OH-D-EQ production between two and three times with the presence of DDRC. The highest level of 5-OH-D-EQ was produced by *L. fermentum* INIA 584L and *L. fermentum* INIA when pNZ:TuR.ifcA was incorporated, with concentrations close to 70 μM of 5-OH-D-EQ.

**Figure 6:**
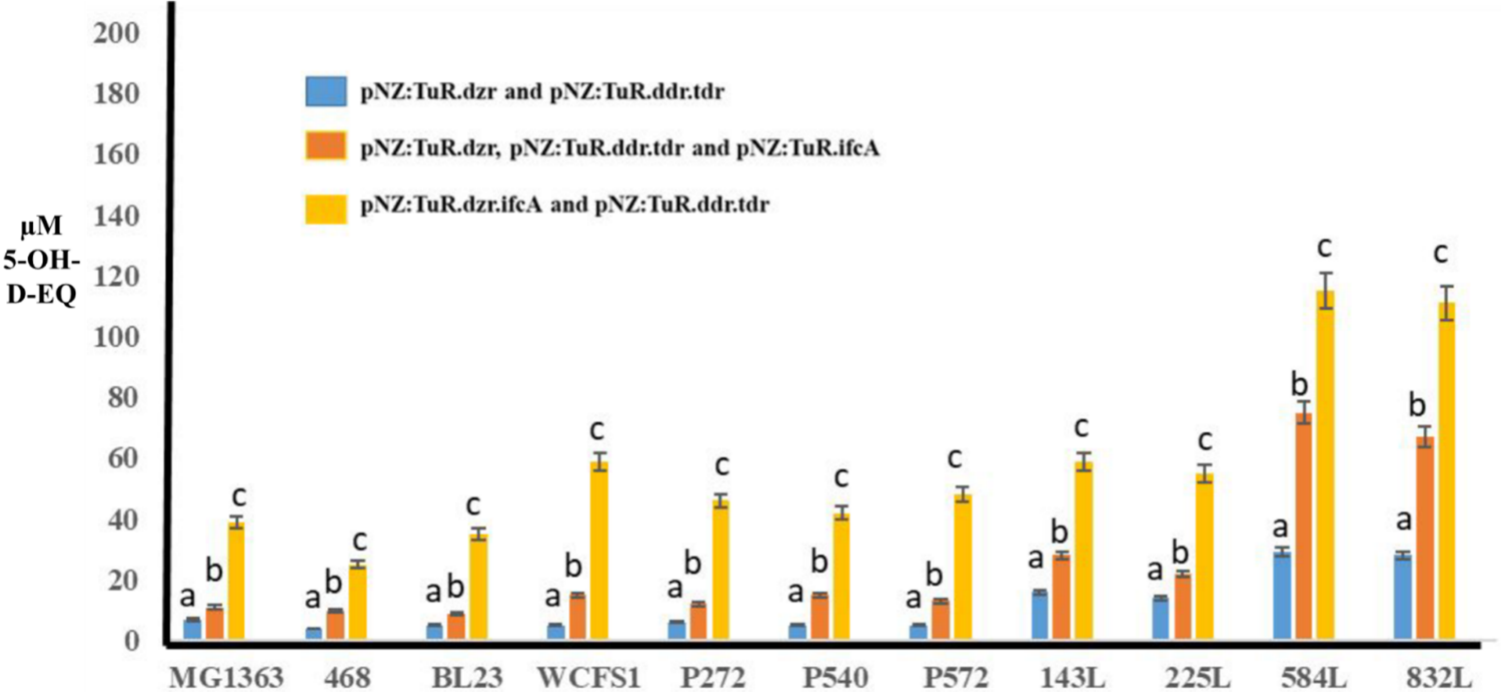
5-hydroxy-dehydroequol (5-OH-D-EQ) production from genistein after 72 h by LAB strains harboring the following combinations of plasmids: (i) pNZ:TuR.dzr and pNZ:TuR.tdr.ddr; (ii) pNZ:TuR.dzr, pNZ:TuR.tdr.ddr and pNZ:TuR.ifcA; (iii) pNZ:TuR.dzr.ifcA and pNZ:TuR.tdr.ddr. Different letters indicate significant difference at the level of *p* < 0.01.

### Effect of the Heterologous Expression of *ifc*A and *dzr* in Different Vectors and in the Same Cell in EQ Production by Engineered LAB Strains

Only *L. cremoris* MG1363, *L. paracasei* BL23, *L. plantarum* WCFS1, *L. fermentum* INIA 143L, *L. fermentum* INIA 584L and *L. fermentum* INIA 849L could be transformed with both plasmids (pNZE.TuR.dzr and pNZ:TuR.ifcA). Figure 1S and Table 4S show how the coincubation of *L. cremoris* MG1363, *L. casei* BL23, *L. plantarum* WCFS1, *L. fermentum* INIA 143L harboring pNZ:TuR.tdr.ddr with these strains transformed with both plasmids (pNZE.TuR.dzr and pNZ:TuR.ifcA), produced a significant (*p* < 0.01) increase in EQ production, compared to the same LAB strains which did not contain ifcA or contained ifcA and dzr in different cells. However, *L. fermentum* INIA 584L and *L. fermentum* INIA 849L harboring pNZE.TuR.dzr and pNZ:TuR.ifcA showed lower equol production than the strains containing *ifc*A (pNZ:TuR.ifcA) and *dzr* (pNZE:TuR.dzr) in different cells. However, the strains harboring pNZE.TuR.dzr and pNZ:TuR.ifcA produced higher concentrations than the same strains without *ifc*A (pNZ:TuR.ifcA). Serial subcultures of these strains showed the stability of different vectors in all the LAB hosts studied for at least 100 generations under nonselective conditions.

### Identification of Translational Coupling in pNZ:TuR.aFP.STOP.mCherry

#### Translation of *mrfp* Occurs Separately, Dependent on the Translation of Evoglow.Pp1

Fluorescence protein expression by *L. cremoris* MG1363, and their transformants harboring pNZ:TuR.aFP, pNZ:TuR.mrfp and pNZ:TuR.aFP.STOP.mCherry, was analyzed by means of native PAGE. The results showed protein bands associated with green fluorescence when these strains were transformed with pNZ:TuR.aFP and pNZ:TuR.aFP.STOP.mCherry (Figure 1). Similarly, protein bands associated with red fluorescence were detected in strains transformed with pNZ:TuR.mCherry and pNZ:TuR.aFP.STOP.mCherry. Protein bands associated with green and red fluorescent in *L. cremoris* MG1363 harboring pNZ:TuR.aFP.STOP.mCherry were separated in the gel and expressed as two independent proteins. Proteomic assays confirmed the independent expression of evoglow.Pp1 and the *mrfp* gene in pNZ:TuR.aFP.STOP.mCherry.

Although evoglow-Pp1 is a smaller protein (19 kDa) than mCherry (26,722 kDa), the molecular weight shown by mCherry in PAGE under native conditions was lower than evoglow-Pp1. This occurs because mCherry is found forming dimers, while aFP is a tetramer. These results coincide with the molecular size of the pattern. Furthermore, denaturation tests using different times showed the presence of mCherry monomers and aFP dimers, although the fluorescence emission was very weak due to denaturation (Data not shown).

To confirm the existence of translational coupling between *evoglow*-Pp1 and *mrfp*, the initial ATG of evoglow.Pp1 was removed from pNZ:TuR.aFP.STOP.mCherry creating the vector pNZ:TuR.aFP.ΔATG.STOP.mCherry. *L. cremoris* MG1363 harboring pNZ:TuR.aFP.ΔATG.STOP.mCherry did not show green or red fluorescent signals (Figure 1). Deletion of the start ATG to prevent translation of Evoglow-Pp1 also prevented the translation of mCherry. Therefore, since the evoglow.Pp1 and mCherry proteins were not produced, no fluorescent signal could be observed.

#### Identification of the Functionality of the Three “ATGs” as Possible Beginnings of *mrfp* Translation

The initial *mrfp* sequence showed the presence of three ATGs in the reading phase (Figure 2A). Blocking two of the three ATGs from the *mrfp* start sequence allowed us to determine that ATG1 of the *mrfp* sequences (pATG1) (with only the initial ATG from the *mrfp* sequence) and ATG3 (pATG3) (only with the third ATG of the *mrfp* sequence) could produce the translation of *mrfp*. pATG3 showed a slightly higher red fluorescence emission compared to pATG1, although this difference was not significant. pATG1 and pATG3 showed a significantly (*p* < 0.01) higher red fluorescence emission compared to pNZ:TuR.aFP.STOP.mCherry, although this was significantly lower than pNZ:TuR.mCherry (Figure 1). Only pATG2 (pNZ:TuR.aFP.STOP.mrfp2) (with the intermediate ATG of the *mrfp* sequence) did not produce a red fluorescent signal emission.

On the other hand, pATG1, pATG2, and pATG3 emitted a green fluorescence signal from the expression of evoglow-Pp1. Moreover, this green fluorescence signal was similar among them, and similar to the green fluorescence signal emitted by pNZ:TuR.aFP.STOP.mCherry. In addition, the green fluorescence signal emitted by these four vectors was between 80 and 90% of the green fluorescence signal emitted by *L. cremoris* MG1363 pNZ:TuR.aFP (Data not shown).

#### Effect of Translational Coupling of *dzr* and *ifc*A on Equol Production

As mentioned above, we demonstrated that the cloned sequence between the stop codon of a fluorescent protein gene (evoglow.PP1) and the ATG of a sequence of another fluorescent protein gene (mCherry) produced translational coupling in pNZ:TuR.aFP.STOP.mCherry, allowing the expression of both fluorescent proteins separately and in an efficient manner (Figure 1). With the aim of improving EQ and analogous compound production, *dzr* and *ifc*A genes were cloned into the same vector (pNZ:TuR.dzr.ifcA) under the influence of the elongation factor Tu promoter from *L. reuteri* CECT925, using the same sequence between *dzr* and *ifc*A that had previously shown translational coupling between evoglowPp1 and the *mrfp* gene (Figure 3).

Except for *L. fermentum* INIA 584L and *L. fermentum* INIA 832L, coincubation of Laboratories harboring pNZ:TuR.dzr.ifcA with the same strains harboring pNZ:TuR.tdr.ddr increased the production of EQ by three to five times when compared to coincubating the strains harboring pNZ:TuR.dzr and pNZ:TuR.tdr.ddr (Figure 4, light orange bars). The incorporation of pNZ:TuR.dzr.ifcA showed an EQ production of 64.50 ± 4.18 μM by engineered *L. cremoris* MG1363, 80.20 ± 7.23 μM by engineered *L. plantarum* WCFS1, and 87.04 ± 8.12 μM by engineered *L. fermentum* INIA 143L. Nevertheless, the translational coupling between the *dzr* and *ifc*A genes did not increase equol production in *L. fermentum* INIA 584L and *L. fermentum* INIA 832L at 72 h. However, the coincubation of both strains harboring pNZ:TuR.dzr.ifcA and pNZ:TuR.tdr.ddr in the presence of daidzein showed an improvement in the efficiency and speed of EQ production, both at 24 and 48 h, with respect to those harboring pNZ:TuR.dzr, pNZ:TuR.tdr.ddr and pNZ:TuR.ifcA strains (Figure 7). The strains that showed translational coupling produced concentrations of EQ close to 100 μM at 24 h, which is double the amount of EQ production of engineered *L. fermentum* INIA 584L and 832L which did not exhibit translational coupling. Serial subcultures of these strains showed stability of pNZ:TuR.dzr.ifcA in all the LAB hosts studied for at least 100 generations under nonselective conditions.

**Figure 7:**
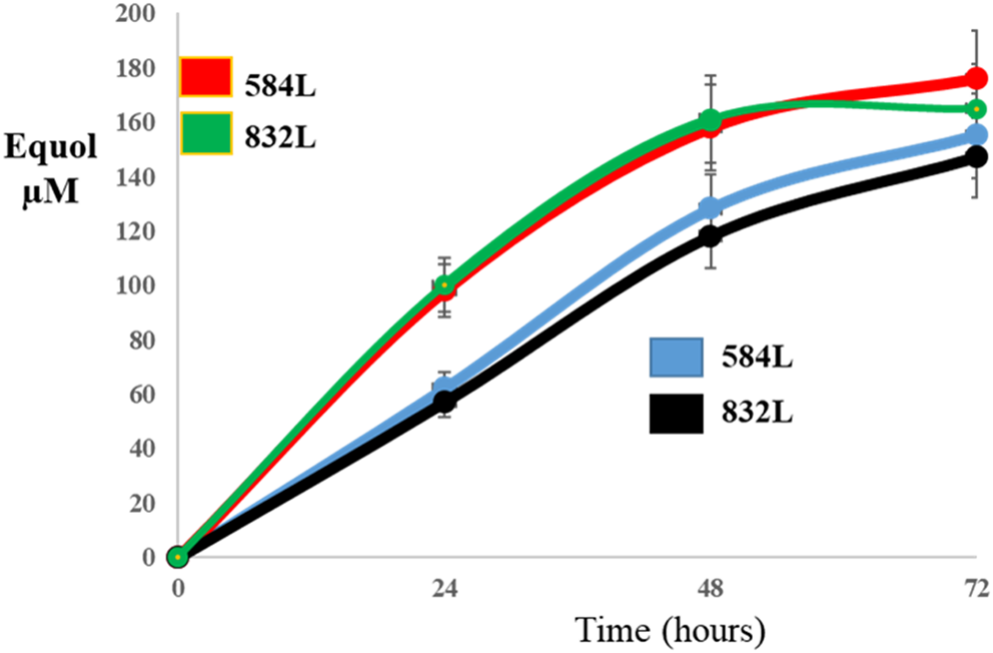
Evolution of EQ production from daidzein by *L. fermentum* INIA 584L and *L. fermentum* INIA 832L harboring the following combinations of plasmids: (i) pNZ:TuR.dzr, pNZ:TuR.tdr.ddr and pNZ:TuR.ifcA (red and green lines) (ii) pNZ:TuR.dzr.ifcA and pNZ:TuR.tdr.ddr (black and blue lines).

#### Effect of Translational Coupling of *dzr* and *ifc*A on 5-OH-EQ Production

Figure 5 (light orange bars) and Table 2S shows how translational coupling of *dzr* and *ifc*A produced a significant effect (*p* < 0.01) on 5-OH-EQ production. Engineered *L. paracasei* BL23, *L. paracasei* INIA P272 and *L. rhamnosus* INIA P540, which had not shown production of this compound with pNZ:TuR.dzr, pNZ:TuR.tdr.ddr and pNZ:TuR.ifcA, now showed production of 5-OH-EQ with the translational coupling of *dzr* and *ifc*A. Concentrations of between 7 and 20 μM of 5-OH-EQ were produced by the engineered Laboratories which showed translational coupling, such as *L. cremoris* MG1363, *S. thermophilus* 468, *L. rhamnosus* INIA P540, *L. plantarum* WCFS, *L. paracasei* INIA P272, *L. paracasei* BL23, *L. reuteri* INIA P572, *L. fermentum* INIA 143L and *L. fermentum* INIA 225L.

Unlike equol production, translational coupling showed a significant (*p* < 0.01) increase in 5-OH-EQ production at 72 h in *L. fermentum* INIA 584L and *L. fermentum* INIA 832L, reaching concentrations higher than 40 μM of 5-OH-EQ (Figure 5 and Table 2S).

#### Translational Coupling Effect of *dzr* and *ifc*A on 5-OH-D-EQ Production

Translational coupling of *dzr* and *ifc*A showed a significant (*p* < 0.01) effect on 5-OH-D-EQ production in all strains (Figure 6 and Table 3S). Translational coupling of *dzr* and *ifc*A showed an increase in 5-OH-D-EQ of up to 10 times with respect a Laboratories harboring pNZ:TuR.dzr and pNZ:TuR.tdr.ddr, and an increase up to 4 times with respect to Laboratories harboring pNZ:TuR.dzr, pNZ:TuR.tdr.ddr and pNZ:TuR.ifcA.

Translational coupling of *dzr* and *ifc*A also showed a significant (*p* < 0.01) increase in the production of this compound at 72 h in *L. fermentum* INIA 584L (115.23 ± 11.55) μM and *L. fermentum* INIA 832L (111.87 ± 30) μM.

### Production of EQ, 5-OH-EQ, and 5-OH-D-EQ in Soy Beverages

To conclude this work, Figure 8 compares the three strategies used with *ifc*A in the production of equol and analogous compounds in soy beverages, that is, with *ifc*A in a vector and an independent cell (pNZ:TuR.ifcA), with *ifc*A and *dzr* in the same cell but in independent vectors (pNZE.TuR.dzr + pNZ:TuR.ifcA), and with translational coupling (pNZ:TuR.dzr.ifcA). We used the strains of *L. fermentum* INIA 584L, *L. plantarum* WCFS1 and *L. paracasei* BL23 with the three vector combinations: (1) pNZ:TuR.dzr, pNZ:TuR.ifcA and pNZ:TuR.tdr.ddr; (2) pNZE.TuR.dzr + pNZ:TuR.ifcA and pNZ:TuR.tdr.ddr, and (3) pNZ:TuR.dzr.ifcA and pNZ:TuR.tdr.ddr. Moreover, *B. pseudocatenulatum* INIA 815 was used in all the combinations and with all the LAB strains for the transformation of daidzin and genistin into their aglycones.

**Figure 8:**
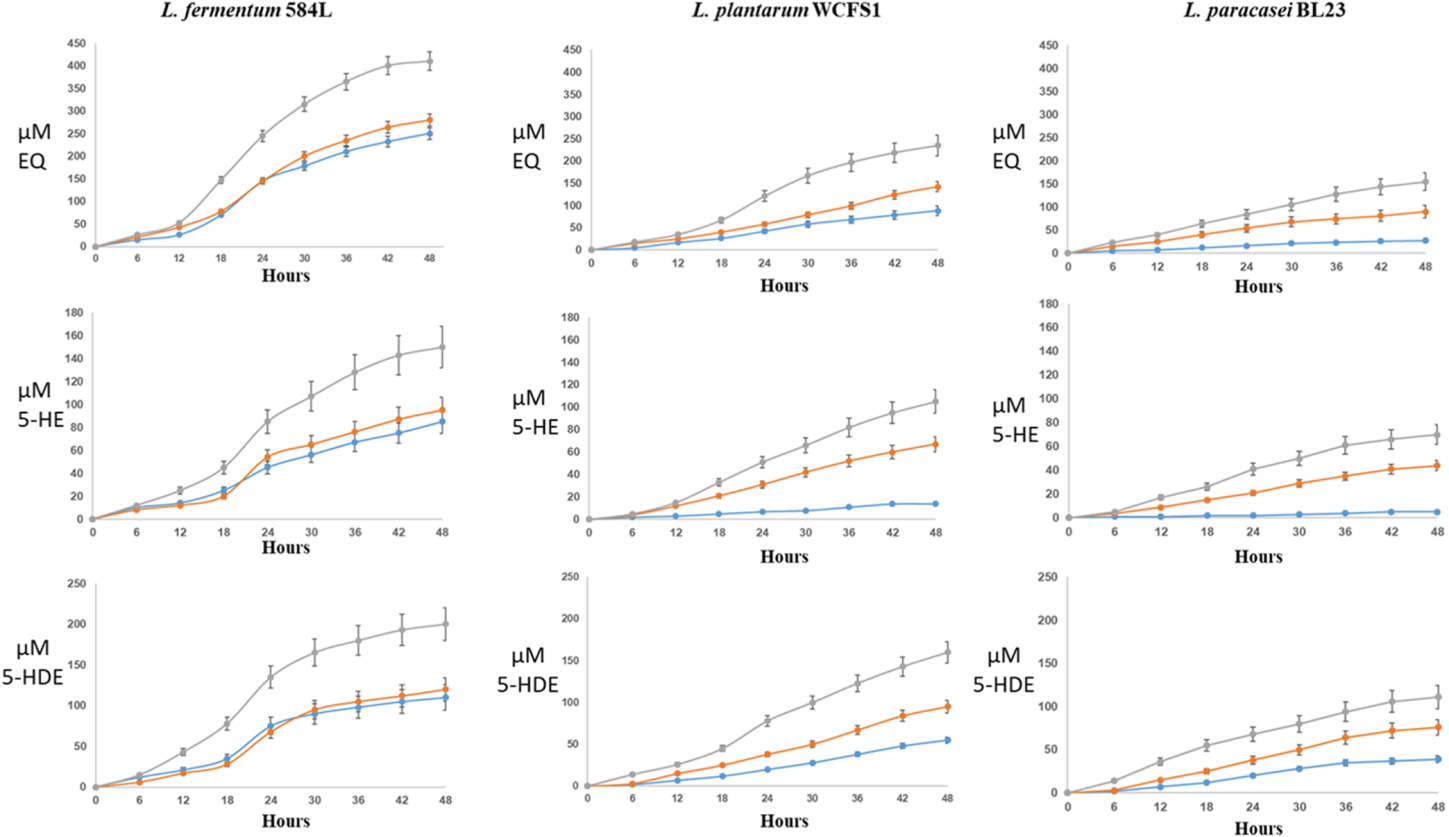
Evolution of production of equol (EQ), 5-hydroxy-equol (5-OH-EQ) and 5-hydroxy-dehydroequol (5-OH-D-EQ) in soy beverages by *L. fermentum* INIA 584L, *L. plantarum* WCFS1 and *L. paracasei* BL23 harboring (1) pNZ:TuR.dzr, pNZ:TuR.ifcA and pNZ:TuR.tdr.ddr (line blue); (2) pNZ:TuR.dzr.ifcA and pNZ:TuR.tdr.ddr (line gray); and (3) pNZE.TuR.dzr + pNZ:TuR.ifcA and pNZ:TuR.tdr.ddr (line orange).

A higher production of EQ, 5-OH-EQ and 5-OH-D-EQ by the strains that presented the translational coupling of the *dzr* and *ifc*A genes (pNZ:TuR.dzr.ifcA) was observed. The combination of *L. fermentum* INIA 584L harboring pNZ:TuR.dzr.ifcA produced 410.56 ± 24.15 μM of EQ, 148.22 ± 9.15 μM of 5-OH-EQ and 201.09 ± 7.65 μM of 5-OH-D-EQ, whereas the combination of *L. plantarum* WCFS1 harboring pNZ:TuR.dzr.ifcA produced 235.77 ± 14.76 μM of EQ, 105.05 ± 11.54 μM de 5-OH-EQ and 161.87 ± 8.34 μM of 5-OH-D-EQ. In addition, the combination of *L. paracasei* BL23 harboring pNZ:TuR.dzr.ifcA produced 135.45 ± 7.08 μM of EQ, 71.00 ± 4.25 μM of 5-OH-EQ and 111.15 ± 9.20 μM of 5-OH-D-EQ (Figure 8).

On the other hand, combination 2 (pNZE.TuR.dzr + pNZ:TuR.ifcA and pNZ:TuR.tdr.ddr) harboring *dzr* and *ifc*A in the same cell, but in different vectors, showed, in *L. plantarum* WCFS1 and *L. paracasei* BL23, a production of equol and analogous compounds lower than that of translational coupling, but much higher than combination 1 which had the *dz*r and *ifc*A in different cells. However, *L. fermentum* INIA 584L did not show significant differences between combinations 1 and 2. In *L. fermentum* INIA 584L strains, combination 2 showed the production of 281.52 ± 14.10 μM of EQ, 95.25 ± 4.89 μM of 5-OH-EQ and 119.96 ± 12.44 μM de 5-OH-D-EQ; in *L. plantarum* WCFS1 strains showed 142.36 ± 17.23 μM of equol, 67.03 ± 5.85 μM of 5-OH-EQ and 95.20 ± 6.15 μM of 5-OH-D-EQ; and *L. paracasei* BL23 strains showed 89.95 ± 3.70 μM EQ, 44.02 ± 8.00 μM of 5-OH-EQ and 76.17 ± 6.84 μM of 5-OH-D-EQ (Figure 8).

On the other hand, combination 1, with *ifc*A in a vector and an independent cell (pNZ:TuR.ifcA), in *L. fermentum* INIA 584L strains showed the production of 247.55 ± 15.33 μM of EQ, 85.17 ± 4.96 μM of 5-OH-EQ and 110.01 ± 7.65 μM of 5-OH-D-EQ; In *L. plantarum* WCFS1 strains, it showed the production of 88.80 ± 5.25 μM of EQ, 14.20 ± 2.17 μM of 5-OH-EQ and 67.94 ± 11.65 μM of 5-OH-D-EQ; and in *L. paracasei* BL23 strains the production of 27.10 ± 1.95 μM of EQ, 5.40 ± 0.45 μM of 5-OH-EQ and 29.66 ± 3.55 μM of 5-OH-D-EQ (Figure 8).

The soy beverages showed initial pHs of 7.04. After 24 h of fermentation, the three combinations of vector in *L. fermentum* INIA 584L strains showed a decrease in pH (5.86 ± 0.26) and at 48 h the pH continued to decrease until reaching a pH of 4.66 ± 0.18. The vector combinations of *L. plantarum* WCFS1 and *L. paracasei* BL23 showed a greater decrease in pH, after 24 h the pHs were 4.95 ± 0.15 and 5.05 ± 0.20, respectively, and after 48 h the pHs were 4.35 ± 0.12 and 4.28 ± 0.15, respectively. We did not observe any significant variations (*P* < 0.01) in the pH between the different combinations of vectors.

The behavior of each strain was studied separately in the soy beverages. All the strains tested for the soy beverage fermentation showed good growth after 24 h incubation, increasing their levels between 1.4 and 2.2 log units, and reaching counts between 8.5 and 9.1 log cfu/mL. Moreover, the three lactobacilli tested maintained their levels over 9 log units after 48 h incubation. Finally, the different combinations of vectors and the presence of two plasmids (pNZE.TuR.dzr + pNZ:TuR.ifcA) in the same cells did not show any significant variations (*P* < 0.01) in the microbial count of three lactobacilli tested.

## Discussion

EQ and 5-OH-EQ are compounds with beneficial effects for human health produced by the intestinal microbiota after the intake of soy.^7,10^ However, most of the Western population cannot produce EQ or 5-OH-EQ,^29^ and the production of these compounds is limited to bacteria that are difficult to grow and unsafe.^7,9,12^ Therefore, the search for safe bacteria able to produce EQ and analogous compounds, which can be easily cultivated and used in food, is of great interest. However, to date, only two strains of Laboratories, *L. fermentum* INIA 584L and *L. fermentum* INIA 832L, harboring the genes involved in EQ production showed a high production of EQ, 5-OH-EQ and 5-OH-D-EQ.^13,14^ Other engineered strains, such as *L. cremoris* MG1363 and *L. plantarum* WCFS1, showed low production of EQ and 5-OH-EQ, while *L. paracasei* BL23 only produced low concentrations of EQ in agreement with previous studies by our group.^14^ New engineered strains of Laboratories producing EQ and analogous compounds were sought in this work. Engineered *S. thermophilus*, *L. paracasei* and *L. rhamnosus* and *L. reuteri* strains showed similar results to those previously observed with *L. paracasei* BL23.^14^ In addition, we included two new strains of *L. fermentum* in order to check whether the high EQ production was related to this species of LAB. Engineered *L. fermentum* INIA 225L and *L. fermentum* INIA 143L showed an EQ production higher that the majority of the engineered LAB strains used in this work, although lower than engineered *L. fermentum* INIA 584L and *L. fermentum* INIA 832L. Moreover, the heterologous expression of *ifc*A did not affect EQ production in *L. fermentum* INIA 225L and *L. fermentum* INIA 143L; however, an effect of the heterologous expression of *ifc*A on 5-OH-EQ production was observed in these strains, something that was only observed in *L. fermentum* strains. On the other hand, all the strains showed an effect of the heterologous expression of *ifc*A on 5-OH-D-EQ production. These results can be explained because 5-OH-D-EQ is produced by the hydration of THG and/or by the higher affinity of DDRC for genistein than for daidzein.^10,13^ Given the only difference between genistein and daidzein is the additional 6-hydroxyl group present in genistein, the presence of the 6-hydroxyl group is important in DDRC activity. In this context, the *O*-demethylase enzyme of isoflavones from *Bifidobacterium breve* INIA P734 managed to transform biochanin A into genistein, but it did not transform formononetin into daidzein,^30^ therefore showing the importance of the 6-hydroxyl group in the enzyme activity. So, DDRC seems to be active in all Laboratories in relation of the increase in the production of 5-OH-D-EQ from genistein, but DDRC does not appear to be active for the production of EQ in most of the engineered LAB strains, since only *L. fermentum* INIA 584L and *L. fermentum* INIA 832L showed an effect of DDRC on EQ production.

As mentioned above, the heterologous expression of *ifc*A resulted in a clear increment in EQ production in engineered *L. fermentum* INIA 584L and 832L, while this effect was not observed in other engineered Laboratories strains. We hypothesize that the effect of DDRC observed in engineered *L. fermentum* INIA 584L and 832L is related to the higher activity of DZR and DHDR presented by these strains, as demonstrated in previous work by our group.^13,24^ Moreover, the greater activity of both enzymes is related to the reducing power.^12^

For years our group has been working on increasing the efficiency of EQ production. So, in order to increase the production of EQ and analogous compounds we previously tried to include the *dzr*, *ddr*, and *tdr* genes in *L. fermentum* INIA 584L in the same cell.^15^ However, EQ production decreased with respect to the expression of *dzr* and *ddr* in different cells. *L. fermentum* INIA 584L harboring the genes *dzr* and *ddr* in the same cell showed high energy consumption and reducing powers because DZNR and DHDR need NADPH as a cofactor.^13,31,32^ The beneficial effect of compartmentalization in EQ production was also described in an *E. coli* *s*train,^10^ and in *S. isoflavoniconvertens*,^14^ when this EQ producing strain exhibited higher production of EQ from DHD compared to daidzein.

In this work, we sought to increase the production of EQ and analogous compounds by working with the heterologous expression of *ifc*A, since the activity of DDRC was necessary to increase the production of these compounds in *L. fermentum* INIA 584L and 832L.^13–15^ Moreover, the effect of DDRC in the production of EQ in *L. fermentum* INIA 584L and 832L harboring pNZ:TuR.ifcA and pNZ:TuR.dzr in separate cells demonstrated that it is not necessary for DZR and DDRC to be in the same cell for the transformation of daidzein into DHD (R) and after into DHD (S). However, the presence of both reactions in the same cell could facilitate the production of EQ. With these results, and since DDRC does not require reducing power for its function, the pNZE:TuR.dzr vector^15^ was transformed into strains harboring pNZ:TuR.ifcA. The presence of both vectors (pNZE.TuR.dzr and pNZ:TuR.ifcA) in the same cell increased EQ production in *L. plantarum* WCFS1 and *L. paracasei* BL23. However, the presence of both vectors did not increase the production of EQ in strains that produced high concentrations of EQ, such as *L. fermentum* INIA 584L and 832L (Figure 1S and Table 4S). Furthermore, many of the strains could not be transformed with both plasmids due to possible interplasmid competition. Later, we hypothesized that the translational coupling of *dzr* and *ifc*A would allow the production of both DZR and DDRC, at similar levels, in order to achieve the production of EQ and analogous compounds efficiently by engineered LAB strains. Moreover, the presence of both *dzr* and *ifc*A genes in the same cell and in the same vector could facilitate the production of EQ and analogous compounds. Therefore, we used a pNZ:TuR vector harboring two fluorescent protein genes together with a stop codon between both genes (pNZ:TuR.aFP.STOP.mCherry)^17^ to achieve our goal.

Our first objective with pNZ:TuR.aFP.STOP.mCherry was to confirm the translation between both fluorescent protein genes. LAB strains harboring pNZ:TuR.aFP.STOP.mCherry showed the emission of green and red fluorescent signals as a consequence of the production of the evoglow.Pp1 and mCherry proteins.^17^ The removal of the initial ATG of *evoglow*.Pp1 (ORF1) did not allow the production of both evoglow.Pp1 and mCherry proteins, and confirmed the translational coupling by demonstrating that the translation of *mrfp* (ORF2) depends on the translation of the gene that precedes it (*evoglow*.Pp1, ORF1). Moreover, the results of the proteomics experiments in this work confirmed that both genes were translated independently, respecting the stop codon between both genes (Figure 1).

Our second objective was to take advantage of the translational coupling of the vector pNZ:TuR.aFP.STOP.mCherry for translational coupling of *ifc*A and dzr, so we built the vector pNZ:TuR.dzr.ifcA. The results shown in Figures 4–8 confirmed the translational coupling and proved our hypothesis that translational coupling would improve the production of EQ and analogous compounds in the engineered LAB strains. Therefore, since DDRC seems to be responsible for the production of high concentrations of EQ, and DDRC did not influence the production of EQ and 5-OH-EQ in the most of engineered LAB bacteria,^14^ translational coupling of *ifc*A and *dzr* allowed DDRC to show activity and increase EQ production in LAB belonging to different genera and species.

Besides translational coupling, other factors must be considered to understand the efficient production of EQ and analogous compounds in engineered LAB strains. First, agreeing with previous works,^13,14^ the transformation of DHD into EQ is much more efficient in Laboratories harboring both the *ddr* and *tdr* genes in the same vector. Hence, the *tdr* and *ddr* genes were cloned into the pNZ:TuR.tdr.ddr vector^14^ using the same sequence as those maintained in the *S. isoflavoniconvertens* genome, and sequence analysis showed that there is a transcriptional coupling between *tdr* and *ddr*, and that the transcription terminator is downstream of *ddr*. This demonstrates that the transcriptional coupling between *ddr* and *tdr* is important in order to produce both enzymes with similar levels to achieve the production of EQ and analogous compounds efficiently. Second, the results shown by Langa et al.^15^ demonstrate that DZR and DHDR must be in different cells for both reductases to function efficiently because of the high consumption of reducing power. Therefore, the translational or transcriptional coupling of the *dzr* and *ddr* genes would not be efficient.

Finally, different LAB strains harboring vectors displaying transcriptional coupling were tested in soy beverages. The results demonstrate that transcriptional coupling allowed engineered LAB strains such as *L. plantarum* WCFS1 and *L. paracasei* BL23 to produce high concentrations of EQ, 5-OH-EQ and 5-OH-D-EQ. To date, only engineered *L. fermentum* strains were able to produce high concentrations of these compounds,^13,14^ and only these strains could be used to enrich soy beverages with EQ, 5-OH-EQ and 5-OH-D-EQ.^15^ Thus, transcriptional coupling has allowed other engineered Laboratories such as *L. plantarum* WCFS1 and *L. paracasei* BL23 to produce soy beverages with significant (*p* < 0.01) concentrations sufficient to exert a physiological effect, in line with the EQ concentrations suggested by other authors.^33–35^ Furthermore, these molecular tools could be transferred to other bacteria with biotechnological interest for the production of high concentrations of both EQ, 5-OH-EQ and 5-OH-D-EQ, as well as other bioactive compounds.

The experiments using soy beverages (Figure 8) demonstrated that translational coupling increases the production of EQ and analogous compounds in engineered *L. fermentum* INIA 584L, *L. plantarum* WCFS1 and *L. paracasei* BL23. Moreover, the high concentration of isoflavones present in the soy beverages together with translational coupling allowed the production of high concentrations of EQ and analogous compounds by these engineered LAB strains.

Although engineered *L. fermentum* INIA 584L strains still produce more EQ, 5-OH-EQ and 5-OH-D-EQ than other engineered Laboratories, the translational coupling between *dzr* and *ifc*A equalizes these differences with other LAB species and genera. The presence of the different vectors, even the presence of several vectors in the same cell, did not show significant differences in the growth of the strains, something previously demonstrated by our group.^15^ Therefore, it could not explain the differences in the production of these bioactive isoflavones. On the other hand, the results shown by Langa et al.^13^ demonstrated that one of the reasons for the greater production of EQ from engineered *L. fermentum* INIA 584L and 832L is the greater activity of DHDR in these strains. Factors such as the difference in pH (*L. fermentum* INIA 584L showed a significantly lower decrease in the pH of soy beverages), and the reducing power could explain these differences. Further studies are needed to better understand the differences between LAB strains in the production of EQ and analogous compounds.

The high levels of EQ and 5-OH-EQ generated by engineered LAB in soy beverages may be advantageous for human health by lessening the effects of menopause and preventing cardiovascular illnesses and certain types of cancer.^7,13,14,33–35^ Nevertheless, 5-OH-D-EQ is a recently discovered compound and its health effects are unknown. Thus, 5-OH-D-EQ could be used in extensive interventional trials to validate its possibly positive benefits through the use of 5-OH-D-EQ-enriched fermented soy beverages. On the other hand, the concentrations of 5-OH-EQ and 5-OH-D-EQ shown in the work are approximate, since we do not have standards for these compounds. So, the appearance of patterns of 5-OH-EQ and 5-OH-D-EQ would be very interesting for the correct quantification of these compounds.

The results shown in the present work constitute a sustainable and economical way of producing high concentrations of EQ and analogous compounds, from economical substrates such as soy and soy beverages, as well as from soy byproducts such as soy flour, which is very rich in isoflavones. However, in order to put these fermented goods in the marketplace, it will be crucial to either remove the DNA of the EQ-producing bacteria described in this work or immobilize the enzymes DZR, DDRC, THDR and DHDR in food grade supports. Moreover, the results presented in this work make evident that the translational coupling strategy can be used for the production of bioactive compounds and the development of functional foods, nutraceuticals and food supplements of high interest to companies and society.

In summary, translational coupling has allowed engineered Laboratories strains belonging to different genera such as *L. fermentum*, *L. plantarum*, and *L. paracasei* to produce high concentration of EQ, 5-OH-EQ, and 5-OH-D-EQ. These results could be exploited by companies in the functional food and health sectors to develop soy beverages enriched with EQ and analogous compounds which could be of great interest regarding the health of certain population groups, as well as for the production of other bioactive compounds.This work demonstrates that the efficiency in the production of EQ, 5-OH-EQ, and 5-OH-D-EQ by engineered LAB strains can be improved with (i) the compartmentalization of the daidzein reductase and dihydrodaidzein reductase enzymes, both enzymes need the presence of reducing power; (ii) the transcriptional coupling of the *ddr* and *tdr* genes; and (iii) the translational coupling of the *ifc*A and *dzr* genes.

## Supplementary Material



## Notes

Supplementary tables and figures (DOCX)

## Abbreviations

EQ: equol
5-OH-D-EQ: 5-hydroxy-equol (5-OH-EQ) and 5-hydroxy-dehydroequol
DHD: dihydrodaidzein
THD: tetrahydrodaidzein
DHG: dihydrogenistein
THG: tetrahydrogenistein
*dzr*: daidzein reductase gene
*ddr*: dihydrodaidzein reductase
*tdr*: tetrahydrodaidzein reductase
*ifcA*: dihydrodaidzein racemase
DZR: daidzein reductase
DHDR: dihydrodaidzein reductase
THDR: tetrahydrodaidzein reductase
DDRC: dihydrodaidzein racemase
LAB: lactic acid bacteria
GRAS: generally recognized as safe
