# Supplementary material for: The Translational Coupling of Daidzein Reductase and Dihydrodaidzein Racemase Genes Improves the Production of Equol and Its Analogous Derivatives in Engineered Lactic Acid Bacteria
Source: ACS Synth Biol. 2025 Oct 24;14(11):4520–32. doi: 10.1021/acssynbio.5c00532 (PMC12645574; doi:10.1021/acssynbio.5c00532)

**Supplementary tables**

|  | **Equol production (µM)** | | |
| --- | --- | --- | --- |
|  | **pNZ:TuR.dzr and pNZ:TuR.tdr.ddr** | **pNZ:TuR.dzr, pNZ:TuR.tdr.ddr and pNZ:TuR.ifcA** | **pNZ:TuR.dzr.ifcA and pNZ:TuR.tdr.ddr** |
| **MG1363** | 21.34 ± 1.25^a^ | 20.73 ± 3.10^a^ | 64.45 ± 8.21^b^ |
| **468** | 11.55 ± 1.15^a^ | 10.32 ± 0.87^a^ | 49.32 ± 5.13^b^ |
| **BL23** | 18.07 ± 2.12^a^ | 20.37 ± 1.14^a^ | 44.40 ± 5.00^b^ |
| **WCFS1** | 19.87 ± 2.87^a^ | 17.53 ± 2.12^a^ | 80.31 ± 7.13^b^ |
| **P272** | 7.05 ± 0.80^a^ | 8.50 ± 1.05^a^ | 35.56 ± 4.78^b^ |
| **P540** | 5.45 ± 0.35^a^ | 6.18 ± 0.95^a^ | 28.07 ± 4.15^b^ |
| **P572** | 10.17 ± 1.05^a^ | 11.22 ± 1.53^a^ | 47.51 ± 3.85^b^ |
| **143L** | 21.09 ± 1.67^a^ | 20.90 ± 2.66^a^ | 87.53 ± 9.11^b^ |
| **225L** | 17.55 ± 2.23^a^ | 18.95 ± 1.96^a^ | 70.42 ± 5.41^b^ |
| **584L** | 39.83 ± 3.57^a^ | 155.42 ± 10.25^b^ | 171.23 ± 14.12^b^ |
| **832L** | 37.42 ± 4.26^a^ | 147.00 ± 12.20^b^ | 165.32 ± 13.32^b^ |

**Table 1S.** Equol production from daidzein after 72 hours by LAB strains harbouring the following combinations of plasmids: i) pNZ:TuR.dzr and pNZ:TuR.tdr.ddr; ii) pNZ:TuR.dzr, pNZ:TuR.tdr.ddr and pNZ:TuR.ifcA; iii) pNZ:TuR.dzr.ifcA and pNZ:TuR.tdr.ddr. Different letters indicate significant difference at the level of p < 0.01.

|  | **5-OH-EQ production (µM)** | | |
| --- | --- | --- | --- |
|  | **pNZ:TuR.dzr and pNZ:TuR.tdr.ddr** | **pNZ:TuR.dzr, pNZ:TuR.tdr.ddr and pNZ:TuR.ifcA** | **pNZ:TuR.dzr.ifcA and pNZ:TuR.tdr.ddr** |
| **MG1363** | 2.30 ± 0.12ª | 2.17 ± 0.12ª | 8.15 ± 1.14^b^ |
| **468** | 3.22 ± 0.45ª | 4.65 ± 0.76ª | 11.07 ± 2.07^b^ |
| **BL23** | n.d.^a^ | n.d.^a^ | 7.03 ± 0.54^b^ |
| **WCFS1** | 1.05 ± 0.17ª | 1.45 ± 0.30ª | 13.66 ± 2.24^b^ |
| **P272** | n.d.^a^ | n.d.^a^ | 10.06 ± 1.04^b^ |
| **P540** | n.d.^a^ | n.d.^a^ | 11.00 ± 0.15^b^ |
| **P572** | n.d.^a^ | n.d.^a^ | 20.34 ± 3.07^b^ |
| **143L** | 4.33 ± 0.55ª | 8.22 ± 1.00^a^ | 20.45 ± 1.67^b^ |
| **225L** | 5.65 ± 0.37ª | 7.05 ± 0.98ª | 17.12 ± 1.03^b^ |
| **584L** | 9.45 ± 1.06ª | 16.71± 1.87ª | 43.40 ± 5.56^b^ |
| **832L** | 10.56 ± 1.78ª | 19.09 ± 2.16ª | 41.25 ± 6.10^b^ |

**Figure 2S.** 5-hydroxy-equol (5-OH-EQ) production from genistein after 72 hours by LAB strains harbouring the following combinations of plasmids: i) pNZ:TuR.dzr and pNZ:TuR.tdr.ddr; ii) pNZ:TuR.dzr, pNZ:TuR.tdr.ddr and pNZ:TuR.ifcA; iii) pNZ:TuR.dzr.ifcA and pNZ:TuR.tdr.ddr. Different letters indicate significant difference

at the level of p < 0.01.

|  | **5-OH-D-EQ production** (**µM)** | | |
| --- | --- | --- | --- |
|  | **pNZ:TuR.dzr and pNZ:TuR.tdr.ddr** | **pNZ:TuR.dzr, pNZ:TuR.tdr.ddr and pNZ:TuR.ifcA** | **pNZ:TuR.dzr.ifcA and pNZ:TuR.tdr.ddr** |
| **MG1363** | 7.05 ± 0.45ª | 11.34 ± 2.47^b^ | 39.57 ± 1.86^c^ |
| **468** | 4.20 ± 0.77ª | 10.77 ± 0.55^b^ | 25.15 ± 2.03^c^ |
| **BL23** | 5.02 ± 0.15ª | 9.08 ± 0.11^b^ | 35.79 ± 3.58^c^ |
| **WCFS1** | 5.34 ± 1.20ª | 15.16 ± 1.96^b^ | 59.32 ± 3.51^c^ |
| **P272** | 6.67 ± 1.19^a^ | 1.44 ± 0.65^b^ | 46.57 ± 3.49^c^ |
| **P540** | 5.80 ± 0.67ª | 15.52 ± 2.43^b^ | 43.29 ± 1.80^c^ |
| **P572** | 5.22 ± 1.12ª | 13.76 ± 2.82^b^ | 48.30 ± 1.27^c^ |
| **143L** | 16.47 ± 2.67ª | 28.26 ± 3.74^b^ | 59.13 ± 6.31^c^ |
| **225L** | 14.00 ± 1.30ª | 23.83 ± 3.96^b^ | 55.11 ± 7.34^c^ |
| **584L** | 29.33 ± 4.17ª | 75.36 ± 6.99b | 115.85 ± 14.84^c^ |
| **832L** | 28.07 ± 3.02ª | 67.33 ± 7.09^b^ | 111.36 ± 9.89^c^ |

**Figure 3S.** 5-hydroxy-dehydroaequol (5-OH-D-EQ) production from genistein after 72 hours by LAB strains harbouring the following combinations of plasmids: i) pNZ:TuR.dzr and pNZ:TuR.tdr.ddr; ii) pNZ:TuR.dzr, pNZ:TuR.tdr.ddr and pNZ:TuR.ifcA; iii) pNZ:TuR.dzr.ifcA and pNZ:TuR.tdr.ddr. Different letters indicate significant difference at the level of p < 0.01.

|  | **Equol production (µM)** | | | |
| --- | --- | --- | --- | --- |
|  | **pNZ:TuR.dzr and pNZ:TuR.tdr.ddr** | **pNZ:TuR.dzr, pNZ:TuR.tdr.ddr and pNZ:TuR.ifcA** | **pNZE:TuR.dzr + pNZ:TuR.ifcA and pNZ:TuR.tdr.ddr** | **pNZ:TuR.dzr.ifcA and pNZ:TuR.tdr.ddr** |
| **MG1363** | 21.41 ± 1.29ª | 20.55 ± 6.01^a^ | 54.97 ± 6.29^b^ | 64.13 ± 2.93^b^ |
| **BL23** | 18.34 ± 0.50ª | 20.39 ± 2.69^b^ | 31.34 ± 2.93^b^ | 44.85 ± 1.94^c^ |
| **WCFS1** | 19.08 ± 2.86ª | 17.27 ± 1.67ª | 50.16 ± 4.48^b^ | 80.38 ± 3.57^c^ |
| **143L** | 17.56 ± 4.44ª | 18.39 ± 0.75ª | 46.81 ± 5.21^b^ | 70.46 ± 8.83^c^ |
| **584L** | 39.39 ± 3.10ª | 155.20 ± 12.68^c^ | 124.89 ± 8.25^b^ | 171.77 ± 17.90^c^ |
| **832L** | 37.47 ± 7.69ª | 147.80 ± 9.67^c^ | 115.16 ± 16.27^b^ | 165.7 ± 10.81^c^ |

**Table 4S.** Equol production from daidzaein after 72 hours by LAB strains harbouring the following combinationas of plasmids: i) pNZ:TuR.dzr and pNZ:TuR.tdr.ddr; ii) pNZ:TuR.dzr, pNZ:TuR.tdr.ddr and pNZ:TuR.ifcA; iii) pNZE:TuR.dzr + pNZ:TuR.ifcA and pNZ:TuR.tdr.ddr; iv) pNZ:TuR.dzr.ifcA and pNZ:TuR.tdr.ddr. Different letters indicate significant difference at the level of p < 0.01.

**Figure 1S.** Equol production from daidzein after 72 hours by LAB strains harbouring the following combinations of plasmids: i) pNZ:TuR.dzr and pNZ:TuR.tdr.ddr; ii) pNZ:TuR.dzr, pNZ:TuR.tdr.ddr and pNZ:TuR.ifcA; iii) pNZE:TuR.dzr + pNZ:TuR.ifcA and pNZ:TuR.tdr.ddr; iv) pNZ:TuR.dzr.ifcA and pNZ:TuR.tdr.ddr. Different letters indicate significant difference at the level of p < 0.01.


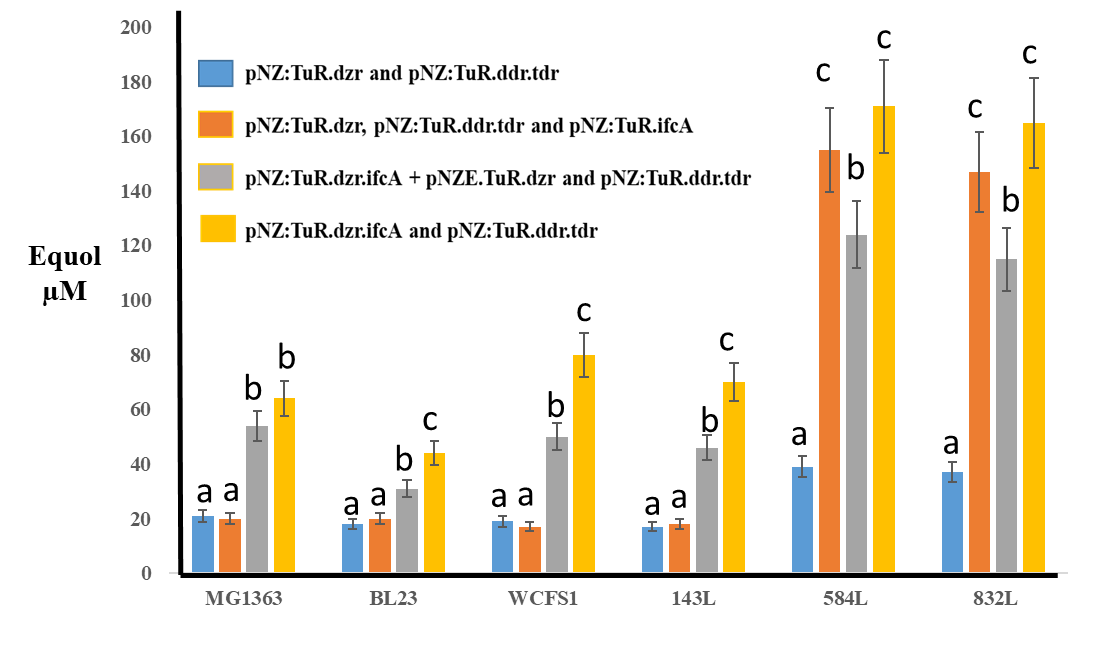

Supplement: Supplementary file 1 [file sb5c00532_si_001.docx]
